# List of Counsel and Witnesses

**Published:** 1852-04-01

**Authors:** 


*Counsel for Petitioners*—Sir F. THESIGER, M.P., and — PETERSDORFF, Esq.

*Counsel for Mrs. Cumming.*

EDWIN JAMES, Esq., Q.C.; Mr. Serjeant WILKINS; F. SOUTHGATE, Esq.

*Solicitor for Petitioners*—Mr. JOHN TURNER.

*Solicitors for Mrs. Cumming*—Messrs. ROBINSON and HAYNES.

### *General Witnesses for the Petitioners.*

Thomas Smith, Clerk to Commissioners of Lunacy.
R. W. T. Lutwidge, Secretary to Commissioners.
H. S. Law, Solicitor to Commissioners.
Elizabeth Brown, servant.
Susan Boyd, ditto.
Harriet Brown, ditto.
Sarah Allsopp, ditto.
George Vernon Driver, surgeon.
Thomas Ince.
Mr. Dangerfield, solicitor.
Winifred Todd, servant.
Mary Rainey, ditto.
James Richards, policeman.
Arthur Parsons, ditto.
Eleanor Hickey, servant.
Mary Ann Hickey, ditto.
Ellen Thompson, ditto.
Simeon Thorne, solicitor.
Nathaniel Webb, land-agent, Newport.
Mrs. Catherine Ince.
Daniel Pilditch, builder.
Benjamin Bailey Hooper.
John Turner, Solicitor for the Petitioners.
James Johnson, policeman.

### *Medical Witnesses.*

George Cornelius Johnson, Esq., surgeon.
Thomas Wilmot, Esq., ditto.
William Bloxam, Esq., surgeon, to produce books of the late Dr. Millingen.
Sir Alexander Morison.
Dr. William King, of Brighton.
Dr. Monro.
Dr. W. V. Pettigrew.
Dr. Hugh W. Diamond.
Dr. J. G. Davey.
Dr. C. J. B. Aldis.

### *General Witnesses for Mrs. Cumming.*

Francis Farrer, solicitor.
Edward Henry Hawkins, a clerk.
Joseph Charles Evans, servant.
Ann Evans, ditto.
John Thomas Stocken, hair dresser.
Matilda Cramer, shopwoman.
Elizabeth Buck, servant.
John Green, policeman.
John James Martin, servant.
John Green, coachman.
Joseph Haynes, solicitor.
John Carlon, ditto.
Francis Francis, coachman.
William Gaywood, ditto.
Elizabeth Clarke, servant.
George Clarke, ditto.
Rev. Chancellor Williams.
Lewis Edmonds, alderman, of Newport.
Thomas Evans, land-agent, dissenting minister.
Thomas George, farmer.
Esther Blake, servant.
Richard Mullock, alderman, of Newport.
Miss Mary Hunt.
Mrs. Eliza Rosina Cooke.
Mr. Leopold Fischel.
James Kell, coachman.
Charles Crane, servant.
Mr. Stephen Hutchinson.
Mrs. Sarah Hutchinson.
Robert Crooke Romsey, solicitor.
James Oldfield, a clerk.
Mr. Charles Ellis.
Albina Watson, servant.
Mrs. Mary Moore.
Jacob Hibbert, builder.
Frederick Lomax, auctioneer.
William Wright Lucking, ditto.
George Chadwin, vestry clerk.

### *Medical Witnesses.*

Dr. Robert Barnes.
Dr. R. J. Hale.
George Simpson, Esq., surgeon.
Dr. Caldwell.
Walter J. Bryant, Esq., ditto.
William Henry Hodding, Esq., surgeon.
Dr. Forbes Winslow.
Dr. Samuel Ashwell.
Dr. John Conolly.
Dr. F. Winslow (recalled).

